# Reviews of Books

**Published:** 1923-05

**Authors:** 


### REVIEWS OF BOOKS.

### THE PSYCHOLOGY OF FEAR.

**Conditions of Nervous Anxiety and their Treatment.** By W. STEKEL. (Kegan Paul; 25s. net.)

This volume, by the well-known Viennese neurologist and psychotherapist, is a most important contribution to medical literature. No better motto could be found for it than that which figures on the title-page, "Behind every thought lurks a craving." The book should appeal to every member of the medical profession. Scientific students of the theory of the unconscious mind will read it with care to ascertain to which particular school of thought the author belongs. He definitely states, in his preface and elsewhere, that he differs from Freud, claiming, in the words of Nietzsche, that his gratitude to the great teacher is indicated by the fact that he is able to go further.

The divergence from Freud's views lies in more than one direction. The point on which the author lays most stress is the identity of neurosis and psychoneurosis. The original view was that the ætiological mechanism of the neuroses was mainly physical, while that of the psychoneuroses was psychical. But this view has been modified by some of Freud's strictest followers, and the theory now held is that the ætiological conditions in the neuroses are derived from comparatively recent life, whereas those of the psychoneuroses belong to infancy. Hence we may all agree with the author in holding that all neuroses are psychical diseases. Again, the question whether every neurosis has a sex basis is disputable. The author repudiates this position. But it is clear, from the clinical histories which he gives, that he traces most of his cases to a sex basis. And the whole dispute turns upon what we are prepared to include under the term "sex," and whether we are ready to use that term in its extended Freudian sense. The author himself admits that the sex-impulse may be directly identified with the instinct of self-preservation. But these and similar questions are really of a somewhat academical nature. It is unreasonable to claim infallibility for the teaching of Freud. Much harm has been done to the cause of mental investigation by the exaggerated claims in this direction which have been made by certain of Freud's more ardent disciples. Freud himself has modified his views in some respects, and so long as we retain his fundamental principle of psychic determinism, there is ample scope for much difference of opinion as to particular applications of his theory. Every science must be progressive, and psycho-analysis, or psychanalysis as Dr. Stekel prefers to call it, can be no exception to this rule.

To the thoughtful general practitioner this book should be of great value. Dr. Stekel points out that the world is full of unnecessary and harmful anxiety. With examples of this condition and of various types of morbid "phobias," he deals in the light of a mass of clinical experience. Who is there who has not had such cases in his practice? And, having eliminated (as Dr. Stekel most carefully insists that we must) all organic causes, have we not dealt with these cases with amused tolerance, with resignation, or with scarcely restrained impatience? Quite ignorant of the real causes of these ills, we have prescribed "tonics" or "change of air and scene," or have counselled our patients to "keep their minds off their troubles and think of something else." And we have been surprised when the patient has deserted us for the faith-healer, the "new-thought" agent, or the Christian Science practitioner. In the light of the rapidly growing literature on this subject, of which this book is an eminent example, there is no longer excuse for such failures, damaging alike to the health of patients and to the reputation of medical men and their profession. We specially commend the perusal of the brilliant chapter on anxiety neurosis in children, as also the author's remarks on the pathological vomiting of pregnancy and the "rudimentary anxiety attack," of which latter condition every practitioner will recall instances such as those described in the book.

But while all should be able to recognise these conditions, it by no means follows that all should attempt their treatment, any more than that all are qualified to carry out ophthalmic or abdominal surgery. The author indicates how wide are the necessary qualifications of a successful practitioner of psychotherapy. Such a man, or woman, needs a full training in neurology and psychiatry, added to some years' experience of general practice. It is also necessary that he should have some knowledge of philosophy, for without this knowledge the study of recent psychological developments is unintelligible, and he should have made a close and sympathetic study of the various forms of religious belief. He needs, in short, to be a "man of the world," in the true sense of that much abused phrase. The author candidly points out certain dangers which will be met with in the practice of psychotherapy, especially as regards the "transference" of the patient's emotions to the analyst. The existence of these dangers has to be faced, although it is no bar to the use of psychotherapy in proper cases and by suitable persons. Psychologists will read with interest the author's discussion of the psychology of fear and anxiety. He maintains the thesis that anxiety is not only a higher degree of fear, but that it also manifests the presence of a repressed instinct. Psychiatrists, too, will find matter for thought in his chapters on the psychic treatment of epilepsy, and on "the border line of psychosis," although they will probably disagree with his view of melancholia.

To the growing body of students of the newer theories of criminality the book should make special appeal. By an extension of our conception of epilepsy, the author would lead us back to the identity of the epileptic and the criminal, a view which was held by Lombroso. Whatever may be our opinions on this point, it is becoming clearer each day how close is the connection between repressed mental conflict and crime. All modern students of criminality are agreed upon this. The author holds that "all neurotics are, in a sense, criminals without the courage to commit a crime." This is too sweeping an assertion. But there is no doubt that there are many instances of what William Healy has called "substitution delinquencies," in which the offence is an attempt at the solution of a mental conflict, although the offence may be of a character apparently quite unconnected with the conflict.

In a final chapter the author discusses the relation of the neuroses to the present organisation of society, and shows how much neurosis is due to repression of the sex instinct. This is, of course, a question of great complexity. We must tread with careful steps. It is easy to say, as the author does, that our civilised sexual morality is not worth the sacrifice which it exacts from us. It is quite another matter to suggest alterations which are at once practicable and unobjectionable. Dr. Stekel most properly and emphatically asserts that he has no intention of advocating a boundless sexual indulgence. But the sane and cautious advance of mental analysis holds out such great hopes for the future that it would be most unfortunate if it should be hindered by any supposed connection with revolutionary ideas as to sex relations. There can be little doubt that harm has already ensued from the exaggerated language which has been used by some ardent advocates. Still it may be that some changes, as for instance in our modes of education of the young in matters relating to sex, are desirable. No progress can be made unless we boldly face these problems. And to all who are prepared to do this the study of the author's conclusions is to be commended.

The translation of the book has been most admirably done by Miss Rosalie Gabler. But we have one final criticism to make, and that a most serious one. The book contains no index—an omission which is little short of a criminal offence.

---

### CHLOROFORM IN THE OPERATING ROOM.

**Chloroform Anæsthesia.** By A. Goodman Levy, M.D.) M.R.C.P. (John Bale, Sons & Danielsson; 7s. 6d. net.

No medical man can afford to have merely vague ideas about chloroform. To anyone may come the startling tragedy of a "death under an anæsthetic." Dr. Levy's views are not yet sufficiently widely known; they are based upon careful experiment and observation, and are deserving of acceptance. While the book is interesting throughout, the chapter in which he maintains that light chloroform anæsthesia is the chief source of danger is most useful. For the reasons advanced in support of this view the book itself must be studied. The use of adrenalin during chloroform anæsthesia is stigmatised as especially dangerous. It is obvious that this is the work of no mere theorist; the mark of the practical man is on every page. Dr. Levy's remarks about silence during the induction of anæsthesia and the importance of restraining the activities of the onlookers who are anxious not to keep the surgeon waiting deserve to be printed in red and framed in every anæsthetising room.

### SIR RAY LANKESTER'S LIGHTER MOOD.

**Great and Small Things.** By Sir Ray Lankester, K.C.B., F.R.S. (Methuen; 7s. 6d. net.)

This is a volume of short scientific essays, originally written for publication in daily and weekly journals. They are twenty-seven in number, and the subjects range from telepathy to the flea parasitic on the pond-snail; or perhaps, as we shall see later, the author would be disposed to reverse this sequence.

Sir Ray Lankester was trained in the school of Huxley and the other great leaders of the Victorian era. To these men the world owes a great debt. Younger people to-day can hardly realise how stifling was the effect of what formerly passed for orthodoxy, and from this the Victorian leaders did much to deliver us. But they did not read the future aright. They looked forward with confidence to a time when the achievements of applied science would transcend anything known before, and when the progress of scientific thought would finally rid the world of the last remnants of ignorance and superstition. Societies were formed, and journals started, for the diffusion of popular scientific knowledge. The first of these anticipations has been amply fulfilled, but not the second. Triumphant democracy seems to care little for that scientific research to which it owes so much; it is supremely suspicious and jealous of scientists, and it ignores scientific work unless the results are served up in sensational form and with due care not to run counter to the pet dogmas of the moment. Superstition, in some respects, appears to be as rampant as ever, and the literature of the day is hardly of a kind to offer encouragement to those who would be hopeful for the intellectual future of the race.

To most of the great men just mentioned, secure as they felt themselves to be in their stronghold of physical science, there was no true psychical activity. To them all psychical existence was consciousness only. Their view of the relation of consciousness to the brain processes held that it was "one of dependence without reciprocity of influence," as Professor McDougall has put it. Sir Ray Lankester does not enter at any length in this book into the problem of the psycho-physical relation. But his position is that which was enunciated by Huxley, and is known as epiphenomenalism. His view as to the consciousness of animals is open to question. He asserts, for example, that a cat is not a reasoning, conscious, being, its behaviour being automatic. Few psychologists and still fewer cat lovers would be disposed to subscribe to this dictum. The modern view is that far less of human conduct, and far more of animal conduct, is due to reason than was at one time held to be the case. Mercier has pointed this out, illustrating it by the example of the spider whose web-making activity is instinctive in origin, but is guided in details by reason.

One of the most interesting Papers is that entitled "Is Nature Cruel?" In this the author deals with the old problem of the existence of pain and other kinds of "evil." He inclines to the view that pain is largely of a protective character, and so may be regarded as a relative good. There are, of course, many other views, and among these may be mentioned that of Spinoza, which holds the relativity of "good" and "evil" to finite and particular interests, the meaning of both terms being transcended in the absolute. Sir Ray Lankester deals drastically with telepathy and kindred subjects, and will have none of them. He points out, very truly, that they have often been mixed up with deliberate fraud. He urges that it is for believers in these matters to bring forward scientific evidence of the reality of the phenomena which they claim to have observed. We still await the production of such evidence. Meanwhile it is well to remember that many scientists of undoubted distinction have expressed the view that physical science does not compel us to hold that the life processes of organisms are completely explained in mechanical terms. The book is written most simply and clearly, and contains some excellent diagrams. It will be read with pleasure by those who enjoy short dissertations on popular science, and it is likely to enjoy as great a vogue as similar works by the same author.

### THE RECONSTRUCTION OF HOSPITAL FINANCE.

**The Scottish Voluntary Hospitals.** By Sir GEORGE BEATSON, K.C.B. (John Smith & Co., Glasgow; 6d.)

In this pamphlet Sir George Beatson, who was Chairman of the Scottish Red Cross during the war, analyses the present financial position of the voluntary hospitals, and suggests a solution of their difficulties. Briefly, he says that these arise from the hospitals no longer confining their treatment to necessitous cases; secondly, the high cost of modern surgery; and thirdly, from no adequate financial changes having been made. He therefore proposes that the general maintenance of a hospital should be provided by the public, and that the patients should provide the cost of the provisions, drugs and dressings used by themselves. The former cost, he adds, forms about two-thirds of the expenditure, and the latter one-third. A charge of 1s. 9d. per day for food, and a uniform rate of 10s. for each medical and 2 guineas for each surgical case requiring an operation, would, he thinks, suffice.

He urges that patients should group themselves into "Hospital Treatment Insurance Societies," which would pay the maintenance charges for their members when in hospital, and thinks that such bodies would be much preferable to the workmen's organisations through which the present contribution schemes are managed. On analysis the scheme, which has been criticised in Scotland as unsuitable to provincial hospitals, does not appear to differ so much in practice from our existing arrangements as Sir George maintains. The public continues to subscribe to hospitals which possess so-called insurance schemes and collect charges from non-contributors, though the division of overhead charges and cost per patient is respected less in theory than in fact.

Perhaps the most interesting criticism in the pamphlet of the present system is that which sees a danger in the workmen's collections of the hospitals losing their independence. The proceeds will be devoted to hospital work. Is not the chief distinction between Sir George's scheme and the present system that whereas the Red Cross Society, under the stimulus of war, found it relatively easy to raise enough money to maintain its hospitals, the voluntary hospitals in time of peace are necessarily more haphazard in their efforts to raise money? But to one who had experience of Red Cross work, the division of expense between the Government grant per patient and the Red Cross funds for maintenance must seem a simple means of solving hospital finance.

### BEHIND THE BARS.

**The Experiences of an Asylum Patient.** By RACHEL GRANT-SMITH. With an Introduction and Notes by MONTAGU LOMAX, M.R.C.S. (Geo. Allen & Unwin; 5s. net.)

It is pretty generally agreed that reforms are needed in asylums, and that the time has arrived when the Lunacy Laws should be revised; but to arrive at a satisfactory conception of what requires most urgently to be improved we must have trustworthy evidence of what is wrong with the present system. When we have found this out we must deal with it in a practical way, and not try to achieve immediately ideal schemes. Great care is obviously necessary in weighing the evidence of the insane or of those who, having been out of their wits, have not entirely regained their mental balance. Yet there are people who seem to be prepared to accept any evidence so long as it supports their contentions. They will be able to add Mrs. Grant-Smith's volume of testimony to the heterogeneous mass they have already accumulated. Dr. Lomax gives it his benison and accepts it as a veracious record.

Those who have had more experience of the insane will not be quite so ready in their belief. It is not necessary to impugn the writer's good faith, but it is not impossible that she writes with a bias of which she is unconscious. It is difficult even on the spot, and when evidence can be obtained from a number of witnesses, to adjudicate on complaints made by patients. How much more involved the matter becomes when a long period has elapsed and confirmatory evidence is not available! There is, too, a type of patient whose life is one long protest against everything which does not square with his wishes. If he is of a litigious cast, he is constantly invoking the aid of every judicial authority and grumbling against them if they fail to act according to his ideas. Such patients are so apt to cause trouble among their fellows—even sometimes among the staff—that their life becomes a perambulation from one institution to another, no one place being able to bear with them for long.

Let it be remembered that patients' complaints really are listened to by the authorities—medical men in charge, members of the Board of Control and others—and acted upon if substantiated. Obviously, however, save in exceptional circumstances, it would be neither fair nor expedient to act upon the unsupported testimony of one patient. It would not, as a rule, be done if the complainant were sane; still less is it justified if he is insane. Asylum reform is going on, and conditions are constantly being ameliorated. When, a few years hence, the fly realises that the wheel is moving he will proudly exclaim, "Look what I have done!" In any case, much may be forgiven to those whose intentions are good, although they sometimes cause more disquiet and do more harm than the really perverse.

### A LIEUTENANT OF FLORENCE NIGHTINGALE.

**Sir Henry Stewart Cunningham.** By Margaret M. Verney (Murray; 1[illegible]s. 6d. net.)

"As noble a figure as I have ever seen of an English gentleman" is the description one of his friends gives of Sir Henry Stewart Cunningham, and this little memoir certainly affords us glimpses of a dignified and charming personality. Son of a distinguished Evangelical Vicar of Harrow, he was educated at Harrow and Trinity College, Oxford, and in the early 'sixties settled down in London, combining a small practice at the Bar with the writing of novels and some journalism. This pleasant life did not, however, last for very long. An unlucky speculation in tea lost him his whole fortune, and it was then that his real life-work began. He was given an appointment as Government Advocate and Legal Adviser in the Punjab, and in 1866, accompanied by his sister, he went out to India, where he remained for twenty-one years and saw service under seven Viceroys. In addition to his legal work Sir Henry had much success with his novels, "The Chronicles of Dustypore," a picture of Anglo-Indian society, being especially popular. In 1873 he became Advocate-General at Madras, a city which from a sanitary point of view was in the most shocking condition. His efforts after improvement brought him into contact with Florence Nightingale, and he became one of her faithful friends and lieutenants in India. Their correspondence opened in this characteristic way:—

> Florence Nightingale is informed by her sister, Lady Verney, that Mr. Cunningham wishes to see her before he leaves England with regard to nursing affairs in Madras. She is afraid this information must be incorrect, as her Indian correspondence for the last ten years on the subject of nursing alone would fill this house; and with Madras a room of this house. And its fruitlessness can only be matched by its size. She would have her Madras correspondence ready for Mr. Cunningham to see, only she fears a path could not then be cleared for Mr. Cunningham to enter the room.

The health conditions of Madras were indeed terrible. Cunningham wrote:—

> Drive along the beach and you are sickened at one place and suffocated at another; pass the Fort and you will be confronted by a smell that is, I verily believe, the worst known to mankind, and the unfortunate soldiers have to sleep in it. We have almost the worst death-rate of any city in India.

Delhi was even more unhealthy—at its drainage and water-supply Miss Nightingale had been working for years. Sir Henry also took an active part in the sanitation of Calcutta, and when at length a Government Sanitary Board was established in every province, it was based on a draft prepared by Henry Cunningham, Colonel Yule and Florence Nightingale. Sir Henry subsequently became a Judge of the High Court of Calcutta and was knighted after his return to England. Still active and young at heart, despite his failing sight, he passed into tranquil old age and, tended by his daughter and surrounded by friends, he passed away leaving a very fragrant memory.

### MISCELLANEOUS NOTICES.

### For the Dermatologist.

Skin Diseases: Their Nursing and General Management. By G. Norman Meachen, M.D., B.S. (Lond.), &c. The Scientific Press. 2nd edition. 3s. 9d. post free.—A second edition of this excellent little manual will be welcomed by nurses, especially those who have already proved its helpfulness to them in the nursing of skin diseases. It has been carefully revised and brought up to date by the author, and a new chapter has been added on the cutaneous manifestations of syphilis. There are also new sections upon occupation-dermatitis, the endocrine glands and on skin disease in school children, which are extremely opportune in the light of present scientific research into the possibilities of a more extended use of gland-therapy, and in view of the rapid developments in public welfare work. Medical students, indeed the busy general practitioner as well, may be glad to turn sometimes to the pages of this clearly-written little book for the solving of a knotty point in diagnosis, or the simplification of some difficult problem arising as to the treatment of everyday skin diseases.

### Marylebone and its Hospital.

The Medical History of St. Marylebone. By Major J. R. Ainsworth-Davis. Mitchell, Hughes & Clarke.—This little history of what Major Ainsworth-Davis calls "the Mecca of the profession" was first delivered as an address to the Middlesex Hospital Medical Society, and a good deal of interesting matter has been compressed into the necessarily limited space. In addition to a description of St. Marylebone as it was in the past, there is an account of the foundation of the Middlesex Hospital, with references to some of the famous men connected with its work. It was reorganised in 1746, having been previously known as the Middlesex Infirmary, where discipline was very slack and the apothecary was dismissed for actions "vile and enormous." Keen interest was displayed in a new direction—that "no woman midwife be permitted to act as midwife to this hospital"—and the hospital narrowly escaped being a lying-in institution pure and simple.

### American Surgery and Public Health.

American College of Surgeons, 1923, Chicago. Annual Report of the Surgeon-General of the Public Health Servce of the United States, 1922. Government Printing Office, Washington.—The tenth year-book of the American College of Surgeons is a comprehensive volume of reference, containing in addition to geographical and alphabetical lists of Fellows various reports of committees and matter on hospital standardisation. A list of approved hospitals is given and the surveys of the College have demonstrated that the American hospitals are "receptive to any means of improving their service to the public." The Surgeon-General's report, dated October 13, 1922, states that "both the morbidity and mortality rates have been lower than in previous years." Marked progress was made in the study and development of industrial hygiene and the facilities of the permanent hospitals improved and extended. The inauguration of a series of public health institutes in certain large cities gave a valuable impetus to better health conditions and together with the advance of health work has come a recognition of the need for trained workers.

### Poisonous Plants.

Poisonous Plants of all Countries. By A. Bernhard-Smith. Baillière, Tindall & Cox. 6s.—In the preface to this second edition the author writes: "I have been criticised, regarding a former work, for not naming the toxins of many of the plants. When these principles have been identified, and presently a revised classification made by the botanists, I shall be in a position to make amends in a future edition."

